# Draft Genome Sequence of Hafnia paralvei Strain GTA-HAF03

**DOI:** 10.1128/genomeA.01592-14

**Published:** 2015-02-19

**Authors:** Melissa E. Kohlman, Catherine D. Carrillo, Alex Wong

**Affiliations:** a Department of Biology, Carleton University, Ottawa, Ontario, Canada; b Canadian Food Inspection Agency, Government of Canada, Ottawa, Ontario, Canada

## Abstract

Hafnia paralvei is a Gram-negative member of the *Enterobacteriaceae* family, closely related to the opportunistic pathogen Hafnia alvei. We report here the first draft genome sequence of H. paralvei, from the beef trim isolate GTA-HAF03, consisting of a 5.0-Mbp assembly encoding 4,382 proteins and 90 predicted RNAs.

## GENOME ANNOUNCEMENT

Hafnia paralvei is a potential pathogen of both animals and humans (1). Originally considered a subtype of Hafnia alvei, differences in the sequences of the *ampC* and 16s rRNA genes led to their taxonomic split (2). There is some uncertainty regarding H. paralvei’s pathogenicity. While H. alvei is often found in the bowels of healthy humans (3), it has been associated with diarrhea in children (3) and with a range of infections in patients with other underlying conditions (4). H. paralvei may similarly be an opportunistic pathogen and has been implicated in a case of bacteremia, cultured concomitantly with Enterococcus faecalis (5). Some studies have suggested that H. paralvei is less pathogenic than H. alvei, due to the absence of Shiga-like cytolytic toxins (3). Moreover, it was recently suggested that strains previously identified as pathogenic H. alvei were isolates of the much more pathogenic bacterium Escherichia albertii (1), and this misidentification may also apply to some H. paralvei strains. Given the association of this organism with food, farm animals and agricultural settings, evaluation of the potential pathogenicity of H. paralvei is important in determining the risk of this bacterium to human and animal health.

H. paralvei GTA-HAF03 was recovered from beef trim, and genomic DNA was isolated from cultures grown overnight on nutrient agar (Difco, Becton, Dickinson & Co., Sparks, MD, USA) using the Maxwell 16 Cell DNA purification kit (Promega, Madison, WI, USA). Sequencing libraries were constructed using the Nextera XT DNA sample preparation kit (Illumina, Inc., San Diego, CA, USA) and paired-end sequencing was performed on the Illumina MiSeq platform (Illumina Inc.), using a 500-cycle MiSeq version 2 reagent kit. Sequencing errors were corrected using Quake version 0.3 with a *k*-mer size of 15 (6), and reads were then assembled *de novo* using SPAdes version 3.1.1 (7). The total length of the draft genome sequence was 4,998,224 bp, in 47 contigs, with an *N*_50_ of 161,556. Contigs were annotated with the NCBI Prokaryotic Genome Annotation Pipeline (PGAP) (8), resulting in the prediction of 4,579 genes, including 4,382 coding sequences (CDSs), and 78 tRNA and 9 rRNA sequences. Phylogenetic analysis based on 16S rRNA sequences clustered this genome with published H. paralvei sequences (data not shown), supporting its identification as H. paralvei rather than its close relative H. alvei.

While the H. paralvei GTA-HAF03 genome lacks the proposed H. alvei virulence factors *eaeA*, *fepA*, and cytolytic toxins (1, 9), a number of genes associated with virulence were detected. For example, the adhesion gene *yidE* found in pathogenic strains of Escherichia coli (10), genes encoding a β-lactamase, and an operon with similarity to a Mycobacterium virulence operon were identified. The absence of some of the key virulence factors encoded in H. alvei strains associated with disease may indicate that H. paralvei GTA-HAF03 poses little risk to human and animal health; however, further *in vitro* and *in vivo* studies are required to characterize virulence potential of this organism.

### Nucleotide sequence accession numbers.

This whole-genome shotgun project has been deposited at DDBJ/EMBL/GenBank under the accession number JWGZ00000000. The version described in this paper is the first version, JWGZ01000000.
